# Epidemiological analysis of human papillomavirus and its subtype infections in 36,248 women in Wuhan, China

**DOI:** 10.3389/fpubh.2026.1745604

**Published:** 2026-01-15

**Authors:** Xin Shen, Weina Guo, Cong Yao, Huan Wu, Yun Xiang, Qingjie Meng

**Affiliations:** 1 Department of Laboratory Medicine, Wuhan Children’s Hospital (Wuhan Maternal and Child Healthcare Hospital), Tongji Medical College, Huazhong University of Science and Technology, Wuhan, China; 2 Health Care Department, Wuhan Children’s Hospital (Wuhan Maternal and Child Healthcare Hospital), Tongji Medical College, Huazhong University of Science and Technology, Wuhan, China

**Keywords:** cervical cancer, genotype, human papillomavirus, prevalence, vaccine, Wuhan, China

## Abstract

**Objective:**

We aimed to analyze the distribution and characteristics of human papillomavirus (HPV) and its subtype infections in women in Wuhan, China, with the objective of providing a reference for the prevention and control of HPV infection and cervical cancer.

**Methods:**

We performed a retrospective study of women who visited a gynecology clinic in Wuhan and underwent HPV typing between January 2021 and December 2023. We determined the HPV subtypes by polymerase chain reaction and diversion hybridization and analyzed the epidemiologic characteristics of the women by age and year groups.

**Results:**

Among 36,248 women, 8,796 were positive for HPV infection, yielding a prevalence rate of 24.27% and showing an annual increasing trend. Single infection was the main type of HPV infection, accounting for 16.83% of cases, whereas multiple infections accounted for 7.44%. The prevalence rates of high-risk, low-risk, and mixed high- and low-risk HPV infections were 18.59, 2.66, and 3.01%, respectively. The prevalence rates of HPV infection in different age groups showed a bimodal U-shaped distribution, with the first and second peaks observed in the ≤24 year (21.45%) and the 55–64 year (32.45%) groups, respectively. A total of 21 HPV subtypes were identified. The five most common high-risk HPV subtypes were HPV-52, 58, 16, 53, and 51, with prevalence rates of 6.12, 3.66, 3.44, 2.68, and 2.41%, respectively. The three most common low-risk HPV subtypes were HPV-CP8304, 42, and 44, with prevalence rates of 2.17, 1.37, and 0.89%, respectively. In the ≤24 year group, the three most common subtypes were HPV-52, 16, and 58. In the ≥55 year group, the three most common subtypes were HPV-52, 58, and 16. The nine-valent vaccine had a 15.27% HPV genotype coverage rate. The high-risk genotype rate not covered by the vaccine was 10.44%.

**Conclusion:**

The HPV prevalence rate among women in Wuhan was higher than that in certain developed cities in China. Among the three existing vaccines, the nine-valent vaccine is more suitable for women in Wuhan. It is necessary to further promote the cervical cancer vaccination program among young women. In addition, cervical cancer screening should be strongly advocated in perimenopausal women. The high frequency of high-risk HPV subtypes that are not covered by existing vaccines highlights the urgent need for new vaccine development based on the HPV epidemiology in the local population.

## Introduction

To date, more than 200 subtypes of the human papillomavirus (HPV) have been reported (1). HPV is a double-stranded DNA virus with a closed circular shape that does not have an envelope (2). Among them, more than 40 subtypes are associated with genital tract infections. HPV subtypes are classified on the basis of their carcinogenic risk as either low or high risk. Low-risk HPV (LR-HPV) subtypes are most commonly associated with benign genital lesions (e.g., condyloma acuminatum), and high-risk HPV (HR-HPV) subtypes are linked primarily to cervical intraepithelial neoplasia and cervical cancer (3).

Cervical cancer is the fourth most common cancer and the fourth leading cause of cancer mortality among women worldwide (4). In 2023, the Catalan Institute of Oncology and the International Agency for Research on Cancer reported 109,741 new cases of cervical cancer and 59,060 cervical cancer-related deaths in China (5). The most effective strategy to control cervical cancer and condyloma acuminatum is to prioritize HPV vaccination. China has approved vaccines for three types of HPV. The bivalent vaccine (Cervarix) targets the HPV-16 and 18 subtypes. The quadrivalent vaccine (Gardasil 4) targets the HPV-6, 11, 16, and 18 subtypes. The nine-valent vaccine(Gardasil 9) targets the HPV-6, 11, 16, 18, 31, 33, 45, 52, and 58 subtypes (6). However, these vaccines can only provide protection against a limited number of HPV subtypes and they were developed based on epidemiological data from Western countries (7) In addition, the distribution and prevalence of HPV subtypes can vary across different countries and regions (8, 9). In the United States, for example, HPV-16 and 18 are the most common HPV subtypes. By contrast, in Asia, Europe, and Oceania, HPV-58 and 52; HPV-31 and 33; and HPV-45 and 73, respectively, are the most common HPV subtypes (10). Therefore, understanding the latest HPV subtype distributions and infection characteristics in different regions is crucial for guiding early screening, vaccination, and development of new vaccines for cervical cancer prevention in the targeted population.

We conducted a retrospective study to assess the prevalence and distribution of HPV subtypes among different age-groups of women who visited the gynecology outpatient clinic of Wuhan Children’s Hospital (Wuhan Maternal and Child Healthcare Hospital, China) between January 2021 and December 2023. We developed an epidemiological reference to help treat and prevent cervical cancer and HPV infections.

## Materials and methods

### Study design and participant selection

Between January 2021 and December 2023, we conducted a retrospective study at the gynecology outpatient clinic of Wuhan Children’s Hospital (Wuhan Maternal and Child Healthcare Hospital, China). All procedures in studies involving human participants were performed in accordance with ethical standards and approved by the Ethics Committee of Wuhan Children’s Hospital (No. 2023R078-E02). The Ethics Committee of Wuhan Children’s Hospital granted an exemption from requiring informed consent.

**Figure 1:**
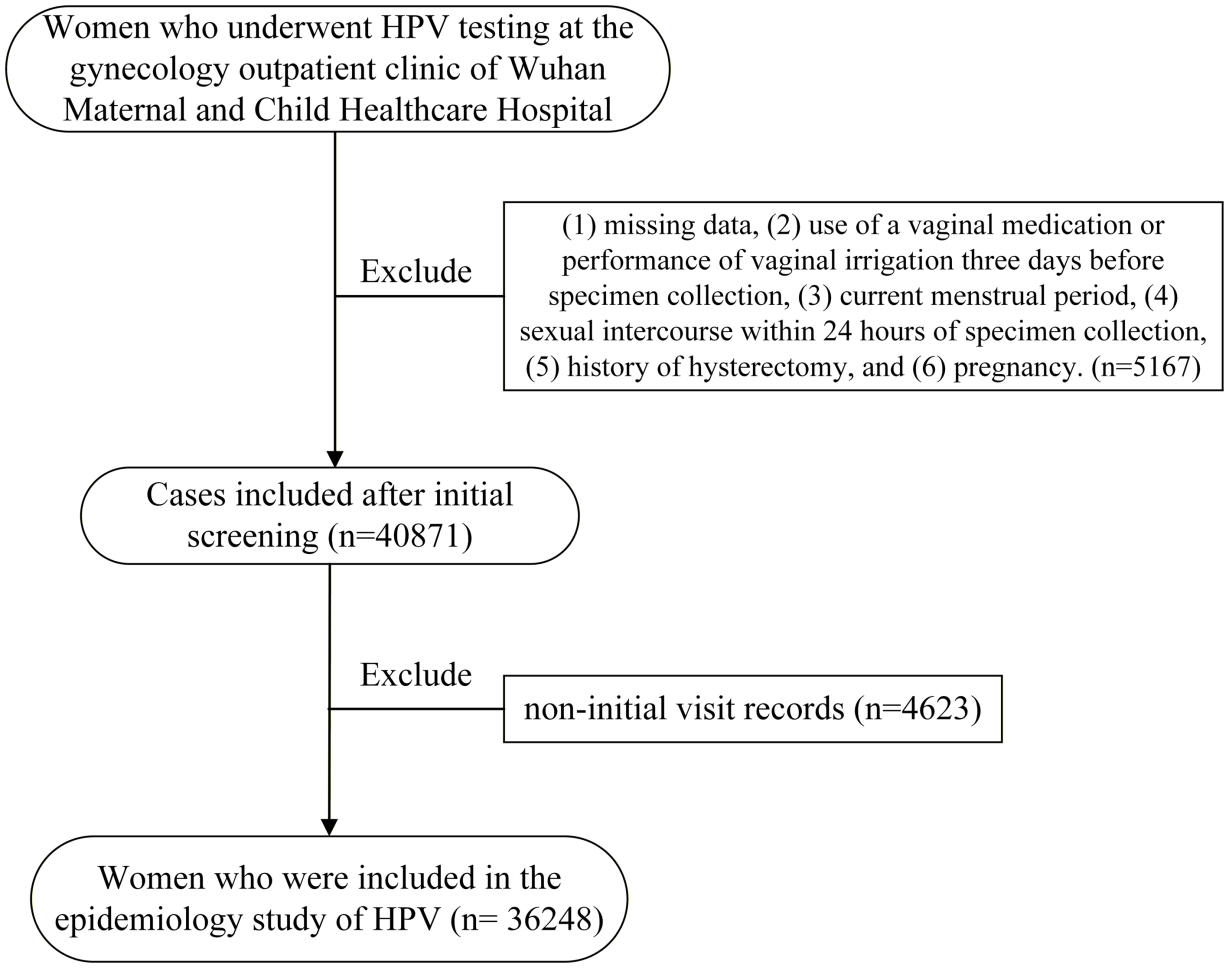
Flow chart of the study participants.

The criteria to participate in the study were as follows: (1) female sex, (2) a resident of Wuhan, (3) had a history of sexual activity and were aged ≥15 years, (4) a complete gynecologic examination, and (5) a complete HPV test. The exclusion criteria were (1) use of a vaginal medication or performance of vaginal irrigation 3 days before specimen collection, (2) current menstrual period, (3) sexual intercourse within 24 h of specimen collection, (4) history of hysterectomy, and (5) pregnancy. For women who had made multiple clinical visits, only the results from the first examination were included. The screening flow diagram is presented in Figure 1.

### Specimen collection

To conduct the gynecologic speculum examination, a cervical brush was inserted into the cervical opening and turned five times in a gentle clockwise fashion. After the brush was carefully removed, it was inserted into a sampling tube. The tube contained a special cell preservation solution and was labeled with a patient number. After it was sealed tightly, the sample was sent for immediate examination or was stored for later examination at 4 °C.

Instruments and reagents HPV subtyping determination was performed by Hybribio Corporation (Guangdong, China). We conducted tests for six low-risk HPV subtypes and 15 high-risk HPV subtypes. The six low-risk subtypes were HPV-6, 11, 42, 43, 44, and CP8304. The 15 high-risk subtypes were HPV-16, 18, 31, 33, 35, 39, 45, 51, 52, 53, 56, 58, 59, 66, and 68. The nucleic acid amplification instrument was purchased from ABI Scientific (Sterling, VA, USA). The HybriMax medical nucleic acid rapid hybridization instrument was purchased from Hybribio Corporation (Guangdong, China).

### DNA extraction

A 200-μL sample was added to columns 1 and 7 of the reagent plate. The deep-well plate containing the samples was then placed in the extraction instrument. One microliter of the eluate from rows 6 and 12 was used as the PCR amplification template.

### PCR amplification and hybridization

One microliter each of the sample DNA, negative control, and positive control from the extraction step was added to the preconfigured PCR mix. The amplification protocol was as follows: (1) 95 °C for 9 min; (2) 95 °C for 20 s, 55 °C for 30 s, 72 °C for 30 s, repeated for 40 cycles; and (3) 72 °C for 5 min.

The PCR products were denatured at 95 °C for 5 min and then immediately cooled in an ice bath for 2 min. The corresponding reagents were added according to the manufacturer’s instructions, and hybridization was performed using a fully automated nucleic acid molecular hybridization instrument. Results were considered valid if the negative control was negative and both the biotin and IC dots were positive. HPV subtypes were identified according to the membrane strip’s HPV gene subtype distribution map.

### Statistical analysis

We used Excel 2016 (Microsoft, Redmond, WA, USA) to record the data, and we used SPSS version 29 (IBM, Armonk, NY, USA) to perform the statistical analyses. We grouped participants according to the following six age-groups: less than or equal to 24 years old, between 24 and 34 years old, between 35 and 44 years old, between 45 and 54 years old, between 55 and 64 years old, and 65 years old or older. To create line charts and bubble plots, we used ggplot2 package of R language (version 4.2.1). Bubble plot was used to display the distribution of high-risk and low-risk HPV subtypes among different age while the overall differences was shown using clustered heatmap. Logistic regression was used to examine the linear annual trend of the positive HPV detection rate. To draw heat maps, we used the ComplexHeatmap package. The distribution of HPV infection subtypes across age groups was visualized using a clustered heatmap. We performed clustering with Euclidean distance as the distance metric. Each block represents the prevalence (normalization by row Z-score after log2-transformed) under specific age group and the corresponding HPV infection subtype. The color of block reflected the magnitude of the deviation. Red represents a higher relative prevalence and gradually turns blue as it declines. We determined statistical significance on the basis of a difference of *p* < 0.05.

## Results

### Overall local epidemiologic characteristics of HPV infections

Among the 36,248 women examined, the overall HPV-positive rate was 24.27% (8,796/36,248). Among the 8,796 HPV-positive cases, single infections accounted for 16.83% of infections and were the main type of HPV infection. Only 7.44% of cases were multiple infections, with dual infections (5.02%) being the main type of multiple infections. Among single infections, the positive rates of high-risk and low-risk infections were 14.34 and 2.49%, respectively. Among multiple infections, the positive rates of high-risk, low-risk, and mixed high- and low-risk infections were 4.25, 0.17, and 3.01%, respectively. Therefore, the highest positive rate among multiple infections was high-risk multiple infections followed by mixed high- and low-risk infections, and the lowest rate was low-risk multiple infections. The subtypes targeted by the bivalent vaccine had a positive rate of 4.48%, the quadrivalent vaccine had a positive rate of 5.49%, and the nine-valent vaccine had a positive rate of 15.27%. The positive rate of the high-risk HPV subtypes not covered by the vaccine was 10.44% (Table 1).

**Table 1:** HPV infection in Wuhan from 2021 to 2023.

Variable	Positive cases, *n*	Prevalence, %	2021 (*n* = 11,654), *n* (%)	2022 (*n* = 8,997), *n* (%)	2023 (*n* = 15,597), *n* (%)
Infection feature					
1	6,100	16.83%	1,598 (13.71%)	1,370 (15.23%)	3,132 (20.08%)
2	1,821	5.02%	390 (3.35%)	405 (4.50%)	1,026 (6.58%)
3	592	1.63%	105 (0.90%)	134 (1.49%)	353 (2.26%)
4	176	0.49%	30 (0.26%)	47 (0.52%)	99 (0.63%)
5	71	0.20%	10 (0.09%)	11 (0.12%)	50 (0.32%)
≥2	2,696	7.44%	538 (4.62%)	602 (6.69%)	1,556 (9.98%)
Types of infection					
Single HR	5,198	14.34%	1,419 (12.18%)	1,171 (13.02%)	2,608 (16.72%)
Single LR	902	2.49%	179 (1.54%)	199 (2.21%)	524 (3.36%)
HR + HR	1,542	4.25%	353 (3.03%)	354 (3.93%)	835 (5.35%)
HR + LR	1,091	3.01%	175 (1.50%)	233 (2.59%)	683 (4.38%)
LR + LR	63	0.17%	10 (0.09%)	15 (0.17%)	38 (0.24%)
Vaccine coverage					
2v	1,623	4.48%	397 (3.41%)	402 (4.47%)	824 (5.28%)
4v	1,989	5.49%	500 (4.29%)	490 (5.45%)	999 (6.41%)
9v	5,534	15.27%	1,382 (11.86%)	1,295 (14.39%)	2,857 (18.32%)
Non-vaccine HR-HPV	3,785	10.44%	893 (7.66%)	803 (8.93%)	2,089 (13.39%)

HPV, human papillomavirus; HR, high-risk; LR, low-risk.

### HPV infection in different age-groups

**Figure 2:**
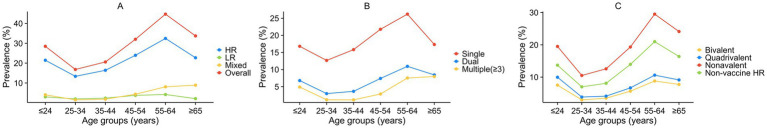
HPV infection rates in different age-groups. **(A)** Low-risk, high-risk, mixed, and overall HPV infection status. **(B)** Single, dual, and multiple infections. **(C)** Bivalent, quadrivalent, and nine-valent vaccine targeted subtypes and non-vaccine high-risk subtypes.

Among the 36,248 women examined, 1,636 were in the ≤24 year group, 13,896 in the 25–34 year group, 10,331 in the 35–44 year group, 5,754 in the 45–54 year group, 4,204 in the 55–64 year group, and 427 in the ≥65 year group. We observed a bimodal U-shaped distribution of HPV infection rates among the different age-groups. The age-group 24 years old or younger had the first peak of HPV infections. The HPV infection rate then dropped, remained stable in the middle age-groups, and then rose to reach the second peak in the 55–64 year group. We observed a similar trend for the high-risk HPV infection rate. The age-group of 24 years old or younger had the first peak (21.45%) and age-group between 55 and 64 years old had the second peak (32.45%). We observed a relatively flat infection curve, however, for the low-risk HPV subtype and the mixed high- and low-risk HPV subtypes (Figure 2A). Among different age-groups, single, dual, and multiple HPV infections showed a bimodal distribution. The rate of infection in the less than or equal to 24 age-group (the first peak) was 16.81% for single infection (275/1,636), 6.78% for dual infection (111/1,636), and 4.89% for multiple infection (80/1,636). The rates of infection in the age-group between 55 and 64 years old (the second peak) were 26.21% for single infection (1,102/4,204), 10.94% for dual infection (460/4,204), and 7.54% for multiple infection (317/4,204), as shown in Figure 2B. The infection rates of the HPV subtypes of the targeted vaccines by age-group also showed a bimodal distribution (Figure 2C).

### Distribution of HPV subtypes

**Figure 3:**
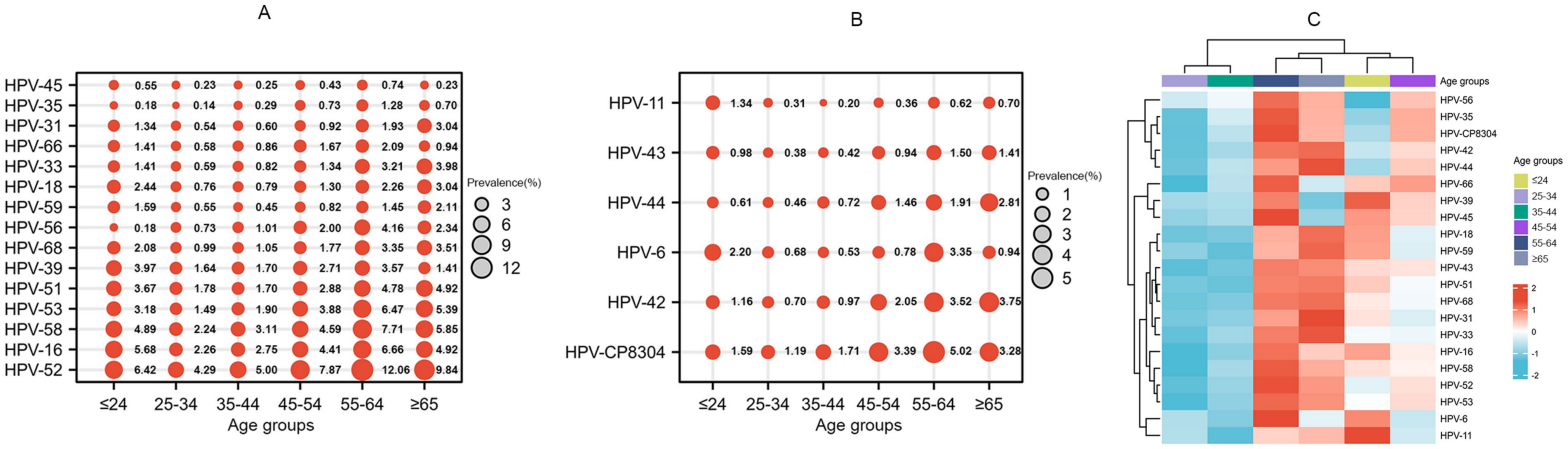
HPV subtype distribution in different age-groups. **(A)** Bubble plots of 15 high-risk subtypes. **(B)** Bubble plots of six low-risk subtypes. **(C)** Heat map of HPV subtype distribution. The positive rate was log-transformed to reduce the skewness of the data.

The positive rates of high-risk, low-risk, and mixed high- and low-risk HPV were 18.59, 2.66, and 3.01%, respectively. The overall positive rate of HPV showed an annual increasing trend (*p* < 0.001). We identified the following five common subtypes of high-risk HPV: HPV-51 (2.41%), HPV-53 (2.68%), HPV-16 (3.44%), HPV-58 (3.66%), and HPV-52 (6.12%). We identified the following three common subtypes of low-risk HPV: HPV-44 (0.89%), HPV-42 (1.37%), and HPV-CP8304 (2.17%). Except for HPV-18, 31, 45, 42, and 11, most subtypes showed significant differences between 2021 and 2023 (*p* < 0.05) (Table 2). The three most common subtypes of low-risk HPV were HPV-6, CP8304, and HPV-11 among the youngest age-group (less than or equal to 24 years old), and the three most common subtypes of high-risk HPV were HPV-52, HPV-16, and HPV-58 among this same age-group (Figures 3A,B). The most common subtypes of high-risk HPV were HPV-52, 58, and 16 among the oldest age-group (55 years old or older), and the most common subtypes of low-risk HPV subtypes were HPV-CP8304, HPV-42, and HPV-6 among this same age-group, as shown in Figures 3A,B. Hierarchical cluster analysis showed that the mid-aged groups (24–34 and 35–44 years) formed one cluster with a similar HPV subtype distribution. The youngest and relatively older groups (≤24 and ≥45 years) formed another cluster with a similar HPV subtype distribution (Figure 3C).

**Table 2:** Distribution of HPV infection subtypes.

HPV subtype	Positive cases, *n*	Prevalence, %	2021 (*n* = 11,654), *n* (%)	2022 (*n* = 8,997), *n* (%)	2023 (*n* = 15,597), *n* (%)	Wald *χ*^2^	*P* ^*^
HPV	8,796	24.27%	2,136 (18.33%)	1,972 (21.92%)	4,688 (30.06%)	349.645	<0.001
HR-HPV	6,740	18.59%	1,772 (15.21%)	1,525 (16.95%)	3,443 (22.07%)	23.692	<0.001
HPV-52	2,220	6.12%	526 (4.51%)	504 (5.60%)	1,190 (7.63%)	77.974	<0.001
HPV-58	1,325	3.66%	323 (2.77%)	301 (3.35%)	701 (4.49%)	35.173	<0.001
HPV-16	1,246	3.44%	289 (2.48%)	303 (3.37%)	654 (4.19%)	48.138	<0.001
HPV-53	973	2.68%	261 (2.24%)	198 (2.20%)	514 (3.30%)	19.02	<0.001
HPV-51	872	2.41%	194 (1.66%)	191 (2.12%)	487 (3.12%)	54.485	<0.001
HPV-39	781	2.15%	219 (1.88%)	179 (1.99%)	383 (2.46%)	7.954	0.019
HPV-56	538	1.48%	87 (0.75%)	109 (1.21%)	342 (2.19%)	72.417	<0.001
HPV-68	537	1.48%	103 (0.88%)	127 (1.41%)	307 (1.97%)	44.499	<0.001
HPV-33	419	1.16%	99 (0.85%)	92 (1.02%)	228 (1.46%)	15.123	<0.001
HPV-18	410	1.13%	114 (0.98%)	105 (1.17%)	191 (1.22%)	3.512	0.173
HPV-66	380	1.05%	90 (0.77%)	73 (0.81%)	217 (1.39%)	15.722	<0.001
HPV-31	306	0.84%	86 (0.74%)	73 (0.81%)	147 (0.94%)	1.217	0.544
HPV-59	266	0.73%	56 (0.48%)	47 (0.52%)	163 (1.05%)	32.296	<0.001
HPV-35	151	0.42%	19 (0.16%)	30 (0.33%)	102 (0.65%)	25.262	<0.001
HPV-45	124	0.34%	29 (0.25%)	36 (0.40%)	59 (0.38%)	3.509	0.173
HR + LR	1,091	3.01%	175 (1.50%)	233 (2.59%)	683 (4.38%)	0.006	0.997
LR-HPV	965	2.66%	189 (1.62%)	214 (2.38%)	562 (3.60%)	0.005	0.997
HPV-CP8304	788	2.17%	163 (1.40%)	155 (1.72%)	470 (3.01%)	51.924	<0.001
HPV-42	498	1.37%	29 (0.25%)	110 (1.22%)	359 (2.30%)	0.004	0.998
HPV-44	321	0.89%	52 (0.45%)	78 (0.87%)	191 (1.22%)	29.837	<0.001
HPV-6	297	0.82%	88 (0.76%)	65 (0.72%)	144 (0.92%)	7.42	0.024
HPV-43	235	0.65%	20 (0.17%)	50 (0.56%)	165 (1.06%)	56.534	<0.001
HPV-11	136	0.38%	38 (0.33%)	34 (0.38%)	64 (0.41%)	3.709	0.157

* Adjusted for age. HPV, human papillomavirus; HR, high-risk; LR, low-risk.

## Discussion

According to the *Global Strategy to Accelerate the Elimination of Cervical Cancer*, which was released in November 2020, the World Health Organization proposed reaching a phased goal of “90–70-90” by 2030. If met, 90% of girls would be fully vaccinated for HPV before they reached 15 years old, 70% of women would be screened with a high-performance test before they reached 35 years old and would be screened again when they reached 45 years old, and 90% of women diagnosed with precancerous lesions or cervical cancer would receive management and treatment that had been standardized (11).

The mortality associated with cervical cancer as well as the incidence of cervical cancer have risen in China since the start of the twentieth century (12). Stopping this rise by preventing and controlling cervical cancer in China requires epidemiological investigation of HPV in China, which was the aim of this study. Between January 2021 and December 2023, the overall prevalence rate of HPV was 24.27% in Wuhan, which is similar to that reported in Chengdu (23.28%) (5) but higher than that reported in developed cities such as Shanghai (18.81%) (13) and Guangzhou (19.78%) (14). The prevalence rates of low-risk HPV and high-risk HPV were higher than the prevalence rate in Guangdong, which was 2.36% for low-risk HPV and 15.66% for high-risk HPV (15). The rate of HPV infection showed significant regional differences, which might be related to different economic levels, lifestyle habits, prevention awareness, detection method sensitivity, and populations included in the study. Women in Wuhan had a relatively high prevalence rate of HPV. This high rate has demonstrated the urgency of increasing the rate of HPV vaccines. It is also essential to expand HPV screening coverage and to minimize cervical cancer incidence among the local population.

As health awareness has increased and as HPV vaccination has become more widespread, previous research has noted a downward trend in the positive rate of HPV (16). However, we found an overall annual upward trend in the HPV-positive rate between 2021 and 2023,which was consistent with the findings of previous studies in Wuhan from 2021 to 2022, despite distinct study populations (17). In a city-wide initiative to enhance women’s health, Wuhan has been providing free cervical cancer screening to all urban and rural women aged 35–64 from 2022 to 2024 (18). This extensive screening program is likely the primary reason for the observed increase in the positive rate. This increase underscores the need for national HPV screening in China. Besides, the observed trend may be related to lifestyle and behavioral changes before and after the COVID-19 pandemic. During the pandemic, strict isolation measures and social distancing effectively reduced the spread of HPV (11). In addition, rates of sexually transmitted infections, including chlamydia, syphilis, and gonorrhea, fell during this time (19, 20), and rates of seasonal respiratory virus infection also decreased significantly (21). Given the opportunity for HPV infections to have increased quickly post-pandemic (22), HPV vaccination programs and cervical cancer screenings are essential to stop HPV infections from continuing to spread.

Among the different age-groups, the prevalence rate followed a bimodal distribution. This result was consistent with the previous studies completed in Chengdu, which showed peaks in the age-groups younger than 20 years old and between 60 and 69 years old (5); in Hangzhou, which showed peaks in age-groups younger than 20 years old and between 61 and 65 years old (23); and in Shanghai, which showed peaks in the age-group younger than or equal to 24 years old and between 55 and 64 years old (13). The prevalence rates among different age-groups for multiple, dual, and single HPV infections aligned with the reported rates for Henan Province (24). The high HPV incidence in women <25 years might be due to their relatively immature immune function, active sexual behavior, and/or limited self-protection awareness. In postmenopausal women, immune dysregulation can impair their ability to effectively clear and suppress the virus, making them more susceptible to persistent HPV infection or the reactivation of latent HPV infection. Therefore, it is necessary to more widely promote cervical cancer vaccination programs among young women and more strongly recommend cervical cancer screening to perimenopausal women. The observed bimodal age distribution in HPV infections in the high-risk but not in low-risk groups was likely due to the fact that high-risk infections are more likely to spread and more difficult to eradicate (25).

It is crucial to understand the distribution of different HPV subtypes in different regions for vaccine development. We identified five of the most common subtypes of high-risk HPV in Wuhan as follows: HPV-52, 58, 16, 53, and 51. By contrast, the five most common subtypes of high-risk HPV in Shanghai were HPV-52, 16, 58, 53, and 39 (13); in Beijing, the five most common subtypes were HPV-52, 58, 16, 51, and 66 (26); and in Yangzhou, the five most common subtypes were HPV-52, 58, 16, 51, and 39 (27). Note, however, that the top three subtypes of HPV were similar across all cities. These subtypes are covered by the nine-valent vaccine, which provides better protection than the bivalent and quadrivalent vaccines. Therefore, among the existing vaccines, the nine-valent vaccine is more suitable for women in Wuhan. The nine-valent vaccine did not cover, however, the common subtypes of high-risk HPV, including HPV-53, 51, 39, and 56. Therefore, future vaccine development should consider these subtypes to provide better health protection for women in Wuhan.

This study has several limitations. Our findings are limited by the use of a clinic-based population, which may overestimate true HPV prevalence due to the higher likelihood of including symptomatic or have abnormal cytology. Given the uneven distribution of age across the study years, we adjusted for age as a potential confounder in our logistic regression models. However, important variables such as HPV vaccination history, parity, contraception, smoking and detailed sexual behavior data were unavailable, which may have introduced residual confounding in the interpretation of the results. Additionally, variations in screening coverage may have influenced the observed trends. In conclusion, the HPV prevalence rate was 24.27% between January 2021 and December 2023 among women in Wuhan and showed an annual upward trend. The most common type of HPV infection remains single infections. This is followed by dual infections and then by multiple infections in frequency. The five most common subtypes of high-risk HPV were HPV-52, 58, 16, 53, and 51. We observed a bimodal U-shaped distribution in the overall HPV prevalence rate across different age-groups. The first peak was observed in the age-group 24 years old or younger. We observed the second peak in the age-group between 55 and 64 years old. The subtype distribution varied by age-group. Hierarchical cluster analysis classified the mid-aged group (25–44 years) into one category and the young/relatively old group (≤24 and ≥45 years) into another category. It is necessary to further promote cervical cancer vaccination programs among young women. In addition, cervical cancer screening should be strongly advocated in perimenopausal women. The development of a multivalent vaccine covering the common HPV-53, 51, 39, and 56 subtypes should be implemented to offer better protection from cervical cancer for women in Wuhan, China.

## Data Availability

The raw data supporting the conclusions of this article will be made available by the authors, without undue reservation.

## References

[1] Zhou L Qiu Q Zhou Q Li J Yu M Li K . Long-read sequencing unveils high-resolution HPV integration and its oncogenic progression in cervical cancer. Nat Commun. (2022) 13:13. doi: 10.1038/s41467-022-30190-1, 35538075 PMC9091225

[2] Bzhalava D Eklund C Dillner J. International standardization and classification of human papillomavirus types. Virology. (2015) 476:341–4. doi: 10.1016/j.virol.2014.12.028, 25577151

[3] Liu M Zhang X Guo L Sun W Jiang X. HPV prevalence and genotype distribution among 38 056 women in Weifang, China: a cross-sectional study. BMJ Open. (2023) 13:13. doi: 10.1136/bmjopen-2023-073332, 37669845 PMC10481741

[4] Sung H Ferlay J Siegel RL Laversanne M Soerjomataram I Jemal A . Global Cancer statistics 2020: GLOBOCAN estimates of incidence and mortality worldwide for 36 cancers in 185 countries. CA Cancer J Clin. (2021) 71:209–49. doi: 10.3322/caac.21660, 33538338

[5] Zhang J Zha T Wang X He W. Prevalence and genotype distribution of HPV infections among women in Chengdu,China. Virol J. (2024) 21:21. doi: 10.1186/s12985-024-02317-x, 38429823 PMC10908056

[6] Zadeh Mehrizi T Ataei-Pirkooh A Eshrati B Ebrahimi SH. Investigating factors affecting the effectiveness of Gardasil 4, Cervarix, and Gardasil 9 vaccines considering the WHO regions in females: a systematic review. Cancer Epidemiol. (2025) 95:95. doi: 10.1016/j.canep.2025.102759, 39914284

[7] Liao G Jiang X She B Tang H Wang Z Zhou H . Multi-infection patterns and co-infection preference of 27 human papillomavirus types among 137,943 gynecological outpatients across China. *Front*. Oncologia. (2020) 10:10. doi: 10.3389/fonc.2020.00449, 32318343 PMC7154087

[8] Zhang J Cheng K Wang Z. Prevalence and distribution of human papillomavirus genotypes in cervical intraepithelial neoplasia in China: a meta-analysis. Arch Gynecol Obstet. (2020) 302:1329–37. doi: 10.1007/s00404-020-05787-w, 32914222 PMC7584548

[9] Derbie A Mekonnen D Nibret E Maier M Woldeamanuel Y Abebe T. Human papillomavirus genotype distribution in Ethiopia: an updated systematic review. Virol J. (2022) 19:19. doi: 10.1186/s12985-022-01741-1, 35033141 PMC8760777

[10] Li N Franceschi S Howell-Jones R Snijders PJF Clifford GM. Human papillomavirus type distribution in 30,848 invasive cervical cancers worldwide: variation by geographical region, histological type and year of publication. Int J Cancer. (2010) 128:927–35. doi: 10.1002/ijc.25396 20473886

[11] Ma Y Xia X Zheng W Dai Y Zhuang X. HPV prevalence and genotype distribution among women in eastern China during the Covid-19 pandemic. Hum Vaccin Immunother. (2023) 19:19. doi: 10.1080/21645515.2023.2212571, 37226673 PMC10294764

[12] Meiwen Y Xuelian Z Honghao W Shangying H Fanghui ZJCEBP. Trend in cervical Cancer incidence and mortality rates in China, 2006-2030: a Bayesian age-period-cohort modeling study. Cancer Epidemiol Biomarkers Prev. (2023):32. doi: 10.1158/1055-9965.Epi-22-0674 36944168

[13] Li X Xiang F Dai J Zhang T Chen Z Zhang M . Prevalence of cervicovaginal human papillomavirus infection and genotype distribution in Shanghai, China. Virol J. (2022) 19:19. doi: 10.1186/s12985-022-01879-y, 36096810 PMC9465878

[14] Li S Zhang K Yang L Wu J Bhargava N Li Y . Distribution patterns of human papillomavirus genotypes among women in Guangzhou, China. Infect Agent Cancer. (2023) 18:67. doi: 10.1186/s13027-023-00541-8, 37907979 PMC10617049

[15] Zhao P Liu S Zhong Z Hou J Lin L Weng R . Prevalence and genotype distribution of human papillomavirus infection among women in northeastern Guangdong Province of China. BMC Infect Dis. (2018) 18:18. doi: 10.1186/s12879-018-3105-x, 29724192 PMC5934871

[16] Yao X Li Q Chen Y Du Z Huang Y Zhou Y . Epidemiology of human papillomavirus infection in women from Xiamen, China, 2013 to 2023. Front Public Health. (2024) 12:12. doi: 10.3389/fpubh.2024.1332696, 38590815 PMC11000419

[17] Guo W Hu Z Yan J Shen X Meng Q Wu H . Epidemiological study of human papillomavirus infection in 105,679 women in Wuhan, China. BMC Infect Dis. (2024) 24:24. doi: 10.1186/s12879-024-10011-0, 39375610 PMC11457396

[18] Wuhan Municipal People's Government. (2024). Free cervical cancer screening for eligible women in Wuhan. Available online at: https://3g.wuhan.gov.cn/hdjl/rdhy/202402/t20240229_2365716.shtml (Accessed Februray 29, 2024).

[19] Tao J Napoleon SC Maynard MA Almonte A Silva E Toma E . Impact of the COVID-19 pandemic on sexually transmitted infection clinic visits. Sex Transm Dis. (2021) 48:e5–7. doi: 10.1097/olq.0000000000001306 33181578 PMC7736141

[20] Ogunbodede OT Zablotska-Manos I Lewis DA. Potential and demonstrated impacts of the COVID-19 pandemic on sexually transmissible infections. Curr Opin Infect Dis. (2021) 34:56–61. doi: 10.1097/qco.0000000000000699, 33315752

[21] Batiha O Al-Deeb T Ea A-z Alsharu E. Impact of COVID-19 and other viruses on reproductive health. Andrologia. (2020) 52:e13791. doi: 10.1111/and.13791, 32790205 PMC7435575

[22] Liu H Yao Q Li D Zhao Z Li Y. Impact of COVID-19 outbreak on the gynecological outpatients HPV infection rate in Wuhan, China: a retrospective observational study. Front Med. (2022) 9:9. doi: 10.3389/fmed.2022.799736, 35479933 PMC9035827

[23] Wang J Zhao K Xia J He F Chen N Wang W . Prevalence and genotype distribution of HPV infection from Hangzhou of Zhejiang Province pre- and during COVID-19 pandemic. *Front*. Public Health. (2024) 12:12. doi: 10.3389/fpubh.2024.1357311, 38873306 PMC11169856

[24] Wang X Song Y Wei X Wang G Sun R Wang M . Prevalence and distribution of human papillomavirus genotypes among women attending gynecology clinics in northern Henan Province of China. Virol J. (2022) 19:19. doi: 10.1186/s12985-021-01732-8, 34991648 PMC8733907

[25] Liu H Wei X Xie Z Wang X Gong X Ke W . Cervical human papillomavirus among 19 753 women attending gynecological department of a major comprehensive hospital in North Anhui China 2013-2016: implication for cervical cancer screening and prevention. J Med Virol. (2018) 91:698–706. doi: 10.1002/jmv.25365, 30475384

[26] Zhu X Wang Y Lv Z Su J. Prevalence and genotype distribution of high-risk HPV infection among women in Beijing, China. J Med Virol. (2021) 93:5103–9. doi: 10.1002/jmv.27013, 33847386

[27] Li Y Liu X Han C Ren C. Prevalence and genotype distribution of high-risk human papillomavirus in 34 420 cases in Yangzhou city, Jiangsu province. China *J Med Virol*. (2021) 93:5095–102. doi: 10.1002/jmv.27012, 33847377

